# Frontline brentuximab vedotin in breast implant‐associated anaplastic large‐cell lymphoma

**DOI:** 10.1002/ccr3.1382

**Published:** 2018-02-16

**Authors:** Juan Pablo Alderuccio, Amrita Desai, Monica M. Yepes, Jennifer R. Chapman, Francisco Vega, Izidore S. Lossos

**Affiliations:** ^1^ Division of Hematology Department of Medicine Sylvester Comprehensive Cancer Center University of Miami Miller School of Medicine Miami FL USA; ^2^ Department of Radiology Sylvester Comprehensive Cancer Center University of Miami Miller School of Medicine Miami FL USA; ^3^ Division of Hematopathology Department of Pathology and Laboratory Medicine Sylvester Comprehensive Cancer Center University of Miami Miller School of Medicine Miami FL USA; ^4^ Department of Molecular and Cellular Pharmacology Sylvester Comprehensive Cancer Center University of Miami Miller School of Medicine Miami FL USA

**Keywords:** Breast implant‐associated anaplastic large‐cell lymphoma and brentuximab vedotin, non‐Hodgkin's lymphoma

## Abstract

We report a woman who developed BIA‐ALCL 9 years after saline implant placement. The lymphoma manifested as a mass lesion associated with axillary lymphadenopathy. She was successfully treated with brentuximab vedotin with minimal toxicity. Brentuximab vedotin may be a promising frontline therapeutic modality for patients with BIA‐ALCL.

## Introduction

Breast implant‐associated anaplastic large‐cell lymphoma (BIA‐ALCL) is a rare T‐cell lymphoma that typically presents as a spontaneous peri‐prosthetic fluid collection or an implant capsular mass 1, 2. The risk factors for developing this disease remain unclear, but both saline and silicone‐filled implants can be associated with BIA‐ALCL 3. The presence of a subclinical biofilm on the implant surface, capsular contracture, repeated capsular trauma, direct toxic damage from the silicone components, and immunologic response has been implicated 4. The median time from implant placement to BIA‐ALCL diagnosis is 9 years (range 1–32), with more than 90% of cases presenting with limited stage lymphoma 5.

There is a lack of treatment standardization for BIA‐ALCL. Histopathologically, lymphoma cells comprising BIA‐ALCL, similar to systemic ALCL, are anaplastic in appearance, are of T‐cell origin, and strongly express CD30 antigen. While brentuximab vedotin, which targets CD30, was demonstrated to be highly effective in treatment of systemic ALCL, 6, 7 to the best of our knowledge, there are no reports on its use as upfront therapy in patients with BIA‐ALCL.

## Case Report

Herein, we present a 57‐year‐old Hispanic woman with a history of left breast ductal carcinoma in situ (DCIS) diagnosed in November 2004. She underwent a left simple mastectomy, prophylactic right mastectomy, and reconstruction with bilateral saline implants. The patient remained asymptomatic until 2009 when she developed bilateral axillary tenderness. She underwent completion of left total mastectomy with left axillary lymph node dissection, revealing stage IA (T1 N0 M0) infiltrating ductal adenocarcinoma, ER+ PR+ Her2 – and DCIS. The patient started adjuvant treatment with docetaxel and cyclophosphamide. After third cycle of chemotherapy, she required hospitalization for shortness of breath secondary to presumptive cyclophosphamide‐induced pneumonitis. Upon recovery, treatment was changed to tamoxifen.

**Figure 1:**
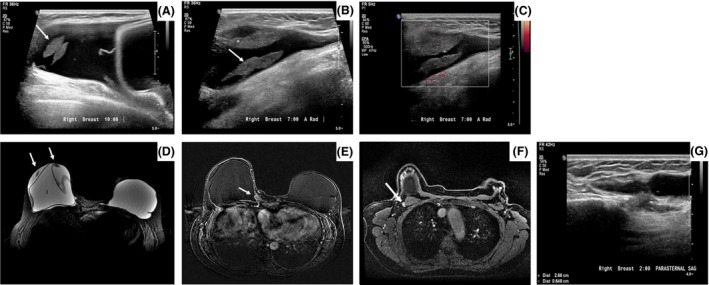
Breast implant‐associated anaplastic large‐cell lymphoma. Radiology. Ultrasound demonstrates a large fluid collection which contains thick septations and solid detritus (arrows) adjacent to the saline implant (A, B), with no evidence of vascularity on Power Doppler imaging (C). Axial MRI STIR sequence obtained in the prone position demonstrates the peri‐implant fluid collection with solid detritus (arrows) surrounding the implant (I) (D). Postcontrast subtraction axial sequence demonstrates a 2.3 cm irregular enhancing mass along the medial aspect of the right implant (arrow) (E). T1 Fat Sat with contrast demonstrates a prominent level 1 axillary lymph node without fatty hilum (F). Second look ultrasound confirmed the presence of the mass that was identified deep to the implant in the supine position (G).

**Figure 2:**
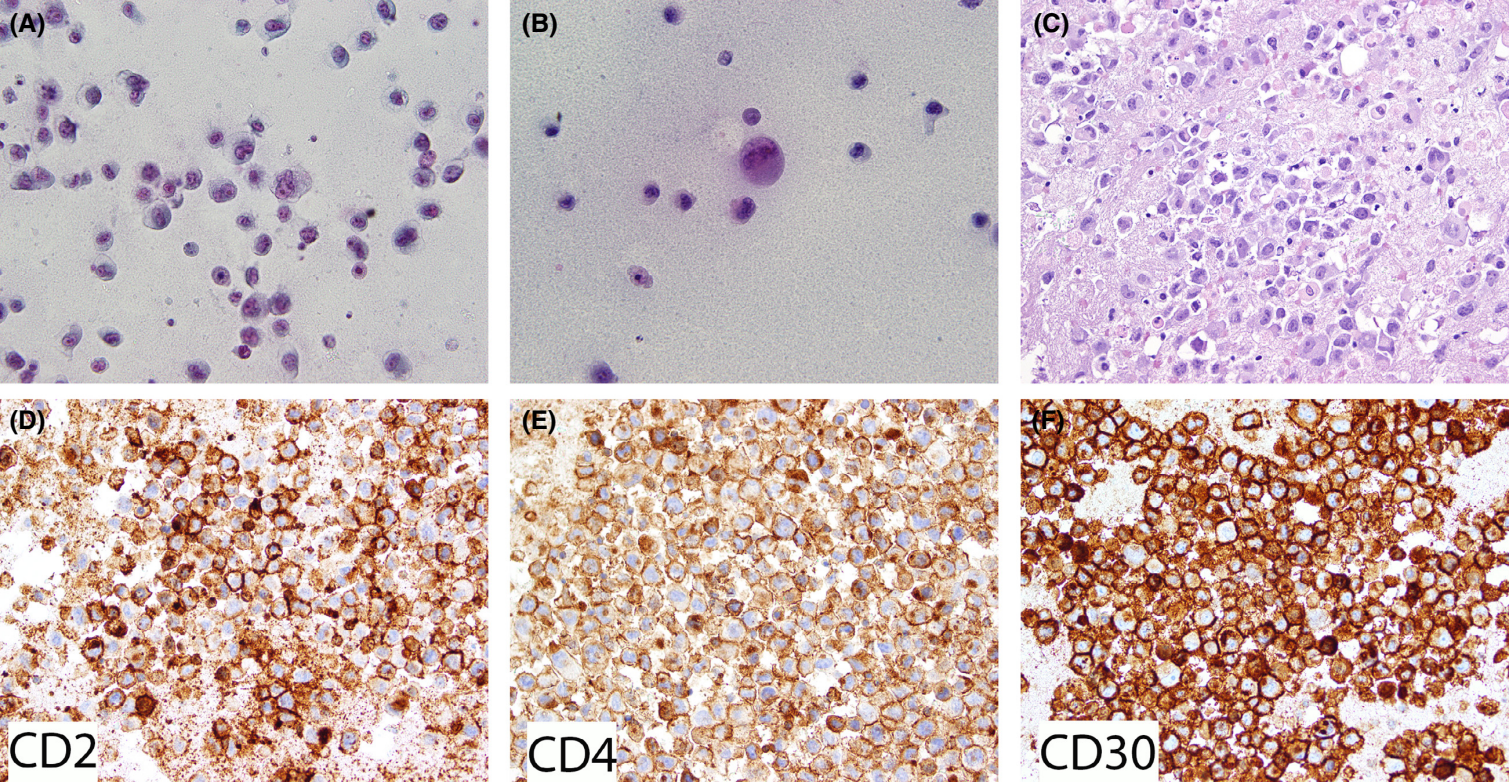
Breast implant‐associated anaplastic large‐cell lymphoma. Pathology. Fine needle aspiration (FNA) of breast implant capsule‐associated fluid (A, B) and cell block specimen prepared from the cells collected by the FNA procedure (C). The cytology and cell block demonstrate a cellular sample containing large, discohesive, cytologically malignant cells, which are characterized morphologically by abundant amphophilic cytoplasm and large nuclei. Nuclear contours ranged from round to significantly indented, and the nuclear chromatin is vesicular with prominent single or multiple nucleoli (A, B, C). Cells with anaplastic morphologic features and hallmark nuclei are frequent. By immunohistochemistry performed in the cell block, lymphoma cells are positive for CD2 (D), CD4 (E), and CD30 (diffuse and strong) (F). Lymphoma cells were also positive for EMA (not shown) and were negative for CD20, PAX5, ALK‐1, TIA‐1, CD3, MPO, CD45, S100, cytokeratin, ER, PR, and HER2 (negative immunostains not shown). Images from panels A‐C were taken from PAP‐stained FNA and cell block samples photographed at 500× original magnification. Figures C–F were photographed at 400× original magnification.

Nine years later, in September 2014, she developed discomfort and swelling in the right breast implant. Ultrasound demonstrated a large fluid collection surrounding the entire implant with avascular detritus and thick septations. (Fig. 1A–C). The patient underwent fine needle aspiration (FNA) of the fluid, which showed an abnormally cellular cystic fluid containing a population of large pleomorphic and cytologically malignant cells. Malignant cells were large and discohesive in nature, with abundant amphophilic cytoplasm and large, often eccentric nuclei (Fig. 2A–C). Nuclear contours ranged from smooth and ovoid to markedly indented, and cells with anaplastic morphologic features, including hallmark cells, were frequent. Immunohistochemically, the malignant cells were positive for CD2, CD4, EMA, and CD30 (diffuse and strong), focally positive for CD8, and negative for cytokeratins, ER, PR, Her2, S‐100, CD45, MPO, CD20, PAX5, ALK‐1, and TIA‐1 (Fig. 2D–F). Lymphoma cell proliferative index, assessed by KI67 immunostaining, was 70%. A monoclonal T‐cell population was detected by multiplex TCR gamma PCR. FISH studies for *DUSP22* and *TP63* rearrangements were negative. Subsequent MRI demonstrated fluid surrounding the right breast implant, with abnormal contour and tethering to the capsule at the anterior aspect, irregular nodular contour of the right inferolateral capsule, and an irregular soft tissue with nodularity measuring 1.0 × 2.3 cm in the axial plane, located at the medial inferior edge of the breast implant. Right axillary lymphadenopathy was also identified (Fig. 1D–F). Second look ultrasound confirmed the presence of the breast mass (Fig. 1G).

Bilateral capsulectomies with implant removal did not detect capsule invasion. Bone marrow biopsy was negative for lymphoma involvement, and PET‐CT, performed after surgery, did not reveal distant lymphoma dissemination (TNM stage IIB [T1 N1 M0], Ann Arbor stage II) 8. No lymph node biopsy/excision was performed at the time of diagnosis. Axillary lymph node presented abnormal characteristics by MRI, and the decision was to proceed with treatment without further investigations. In view of the presence of a mass lesion and lymphadenopathy that are associated with higher relapse rate and shorter survival in previous series, 5, 9, 10 the patient was treated with brentuximab vedotin 1.8 mg/kg every 21 days for 6 cycles. The treatment was uneventful, and the patient achieved continuous complete remission (CR), currently lasting 3 years.

## Discussion

Breast implant‐associated anaplastic large‐cell lymphoma is a rare peripheral T‐cell lymphoma that occurs in patients with breast implants. The estimated incidence of BIA‐ALCL in USA is 3 per 100 million cases per year, as per the SEER data report 2. The median age at presentation is 52 years (28–87 years) 5, 11 with a median time from implant to diagnosis of 9 years (1–32 years) 5. Many studies have failed to show an increased risk attributed to implants when compared to the general population 10.

Most patients (84%) present with early‐stage IE disease.^1^ The lymphoma most commonly manifests as a peri‐prosthesis fluid collection (seroma) but may also present as a capsular mass with or without effusion 5, 9, 10. These clinical presentations are attributed to distinct pathological variants: in situ BIA‐ALCL and infiltrative BIA‐ALCL 9. The in situ variant exhibits a malignant lymphoid proliferation confined to the fibrous capsule, with minimal associated inflammatory response and clinically presents as a seroma. In contrast, the infiltrative variant is characterized by infiltration of lymphoma cells into the capsule with a marked inflammatory response, including large numbers of eosinophils and the presence of capsular mass. These clinicopathological variants exhibit different clinical behaviors: The 2‐year OS is significantly shorter in patients with infiltrative BI‐ALCL (capsular mass) compared to in situ BI‐ALCL (seroma) (52.5% vs. 100%, respectively, *P *=* *0.023) 9. This shorter survival was observed despite implant removal followed by CHOP‐like chemotherapy in majority of these patients. Similar findings were observed by Miranda et al. who reported that patients with capsular mass achieve CR in 72% of cases with a median OS (mOS) of 12 years, while seroma patients achieve CR in 93% of cases and mOS is not reached (*P *=* *0.052) 5. The presence of a capsular mass at diagnosis conferred a hazard ratio for relapse/refractory disease of 12.7 (*P *<* *0.001) 10. Advanced stage and lymph node involvement, commonly observed in patients presenting with a mass, also contribute to inferior outcomes 5, 9, 10. Consequently, BIA‐ALCL patients presenting with a mass have a more aggressive course justifying systemic therapy in addition to removal of implants 5. Disease surveillance encompasses clinical follow‐up every 3–6 months associated with chest, abdomen, and pelvis CT scan with contrast every 6 months for 2 years, and then as clinically indicated 1. If there is a suspicion for recurrent disease, PET–CT is indicated.

There is no standardized therapy for BIA‐ALCL, and success of different therapeutic modalities depends largely on the stage at presentation, lymph node involvement, and pathological variant. NCCN guidelines recommend surgical capsulectomy for early‐stage disease and adjuvant chemotherapy for patients with lymph node involvement and advanced stage. However, relapses are observed with all these approaches and novel, less toxic and targeted therapeutic agents are needed.

Brentuximab vedotin is an antibody‐drug conjugate with a chimeric CD30 antibody attached to monomethyl auristatin E (MMAE), a microtubule inhibitor. Promising results have been observed with brentuximab vedotin as frontline therapy in CD30+ peripheral T‐cell lymphomas and ALCL 6, 12. In a phase II trials of relapsed/refractory systemic ALCL cases, brentuximab vedotin therapy led to an overall response rate (ORR) and CR rate of 86% and 57%, respectively, with median CR duration of 13.2 months 6. When used in a frontline setting with chemotherapy, patients with ALCL had an objective response and CR of 85% and 62%, respectively 12.

Our patient received prior cyclophosphamide‐based therapy for breast cancer developing severe pulmonary toxicity. Therefore, CHOP‐based treatment might compromise her respiratory status. Hence, we decided to use brentuximab vedotin that led to a long‐lasting CR.

To the best of our knowledge, this is the first report on the use of brentuximab vedotin in a frontline setting for treatment of BIA‐ALCL. While additional studies are needed, this may be a promising therapeutic modality for patients with BIA‐ALCL that may offer excellent responses with minimal toxicity.

## Authorship

J.P.A and A.D.: wrote the manuscript. I.S.L.: treated the patient and wrote the manuscript. Y.M.M.: prepared radiology imagines and reviewed the text. J.C. and F.V.: prepared pathology imagines and reviewed the text; all authors approved the final manuscript.

## Conflict of Interest

The authors declare that they have no conflicts of interest.
